# How Big of an Effect Do Small Dams Have? Using Geomorphological Footprints to Quantify Spatial Impact of Low-Head Dams and Identify Patterns of Across-Dam Variation

**DOI:** 10.1371/journal.pone.0141210

**Published:** 2015-11-05

**Authors:** Jane S. Fencl, Martha E. Mather, Katie H. Costigan, Melinda D. Daniels

**Affiliations:** 1 Kansas Cooperative Fish and Wildlife Research Unit, Division of Biology, Kansas State University, Manhattan, Kansas, United States of America; 2 U. S. Geological Survey, Kansas Cooperative Fish and Wildlife Research Unit, Division of Biology, Kansas State University, Manhattan, Kansas, United States of America; 3 Department of Geography, Kansas State University, Manhattan, Kansas, United States of America; Pacific Northwest National Laboratory, UNITED STATES

## Abstract

Longitudinal connectivity is a fundamental characteristic of rivers that can be disrupted by natural and anthropogenic processes. Dams are significant disruptions to streams. Over 2,000,000 low-head dams (<7.6 m high) fragment United States rivers. Despite potential adverse impacts of these ubiquitous disturbances, the spatial impacts of low-head dams on geomorphology and ecology are largely untested. Progress for research and conservation is impaired by not knowing the magnitude of low-head dam impacts. Based on the geomorphic literature, we refined a methodology that allowed us to quantify the spatial extent of low-head dam impacts (herein dam footprint), assessed variation in dam footprints across low-head dams within a river network, and identified select aspects of the context of this variation. Wetted width, depth, and substrate size distributions upstream and downstream of six low-head dams within the Upper Neosho River, Kansas, United States of America were measured. Total dam footprints averaged 7.9 km (3.0–15.3 km) or 287 wetted widths (136–437 wetted widths). Estimates included both upstream (mean: 6.7 km or 243 wetted widths) and downstream footprints (mean: 1.2 km or 44 wetted widths). Altogether the six low-head dams impacted 47.3 km (about 17%) of the mainstem in the river network. Despite differences in age, size, location, and primary function, the sizes of geomorphic footprints of individual low-head dams in the Upper Neosho river network were relatively similar. The number of upstream dams and distance to upstream dams, but not dam height, affected the spatial extent of dam footprints. In summary, ubiquitous low-head dams individually and cumulatively altered lotic ecosystems. Both characteristics of individual dams and the context of neighboring dams affected low-head dam impacts within the river network. For these reasons, low-head dams require a different, more integrative, approach for research and management than the individualistic approach that has been applied to larger dams.

## Introduction

Large dams alter native biodiversity in aquatic ecosystems by modifying geomorphic, hydrological, and ecological connectivity [1, 2]. Large dams fragment riverscapes within the Great Plains [3] by regulating streamflows and dampening floods [4]. For small, low-head dams, however, the potential impacts on geomorphic and ecological impacts are infrequently examined and poorly understood. Although the effect of low-head dams likely extends beyond the immediate vicinity of the dam structure, the spatial extent of low-head dam impacts has not been previously measured in the ecological literature, only estimated (e.g., [5, 6, 7]). Unless scientists and managers can distinguish impacted from unimpacted areas adjacent to dams, environmental professionals will be unable to undertake appropriate research or propose effective management actions to evaluate, understand, and remedy potential fragmentation by low-head dams. Here, we use geomorphic paradigms and metrics to test predictions about the longitudinal extent of low-head dam impacts (hereafter the dam footprint) within the Upper Neosho river network, KS, United States of America. The resulting insights on the size of geomorphic impacts, across-dam variation, and context of this variation will fill important information gaps about these small, but abundant, ecological disturbances.

In addition to the 87,000 large dams listed in the US Army Corps of Engineers National Inventory of Dams [8], 2,000,000 low-head dams (< 7.6 m high) are estimated to fragment United States rivers [9]. A large body of literature documents how large dams alter aquatic ecosystems (e.g., [10, 11]), but data on low-head dams are limited [12]. Most low-head dam studies only sampled at one or two dams per study [13]. By virtue of their abundance, small dams may substantially impact flowing aquatic ecosystems either alone or as a basin-wide cumulative impact. Alternatively, if the dam footprint is small or the physical recovery is rapid, the isolated or cumulative spatial impacts of low-head dams could be negligible.

The extant geomorphology literature provides useful guidance on how dams may effect change via altered channel processes, as well as metrics that can be used to quantify the spatial extent of dam impacts. A Web of Science (WOS) search (9 July 2015) on the keywords “geomorph*” and "low-head dam*,” "low head dam*," “lowhead dam*,” "small dam*," “run-of-river dam*,” or “weir*” identified 54 peer-reviewed publications (S1 Table). Sixty-nine percent (N = 37) of the geomorphic literature on low-head dams addressed issues other than physical conditions adjacent to dams and were not considered further. Only 17 papers (31%) documented geomorphic changes occurring around low-head dams (e.g., extent of channel widening, bar formation, depth, and substrate size). We base our geomorphic predictions for the spatial extent of dam footprints from the WOS literature that reveals expected patterns of geomorphic change around low-head dams and underlying processes for these geomorphic changes.

**Fig 1:**
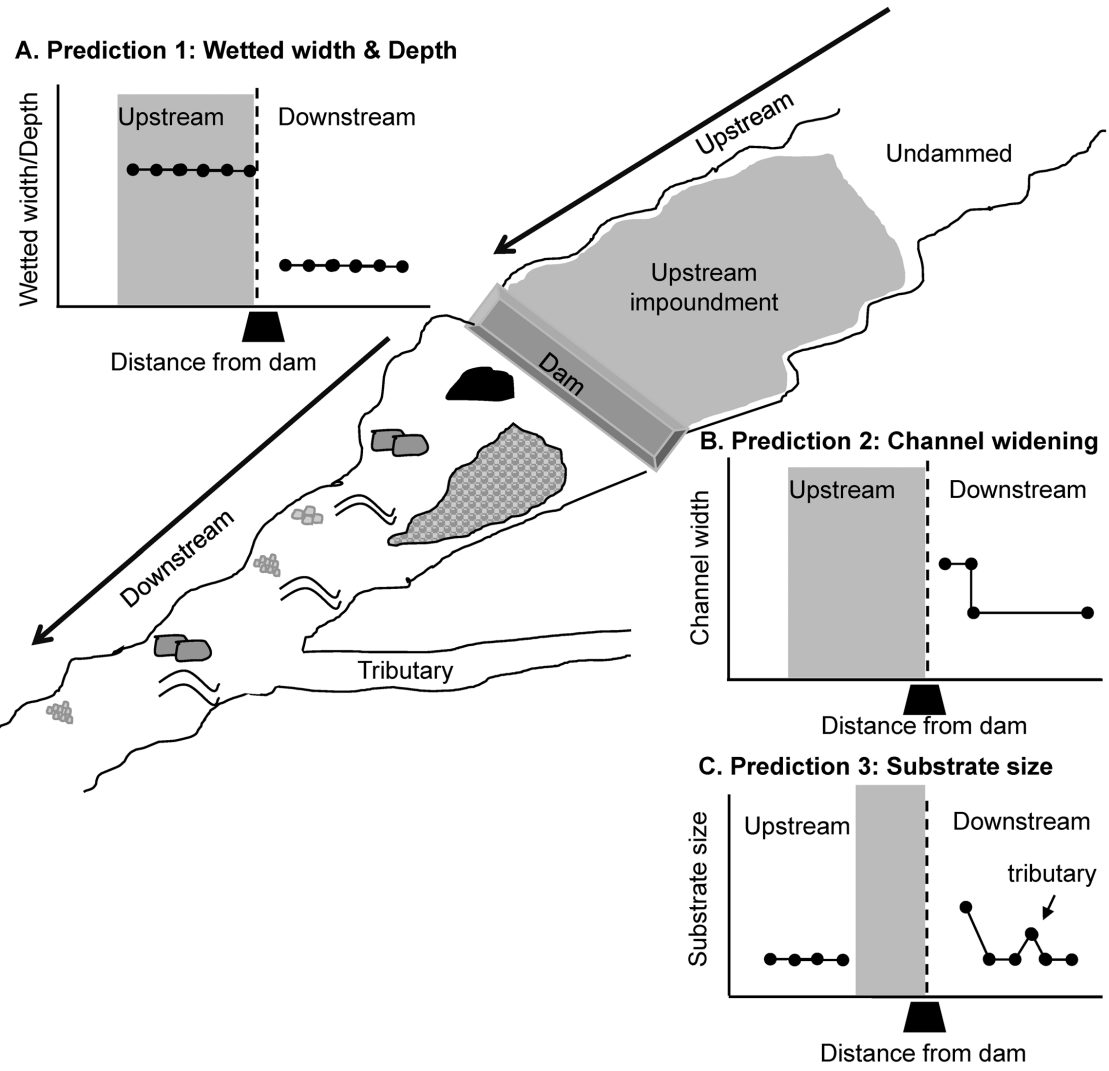
Predictions about Dam Impact. Predictions of geomorphic effects caused by low-head dams on (A) wetted width and depth, (B) channel widening, and (C) substrate size from the Web of Science literature on low-head dams. On all prediction plots, the X axis is the distance from the dam, the black trapezoid represents dam position, the area left of the dashed line represents habitat upstream of the dam and the area right of the dashed line represents habitat downstream of the dam. The impoundment is represented by grey shading.

Specifically, the geomorphic literature predicts three major changes around low-head dams related to dam-induced alterations to flow and sediment regimes (Fig 1). First, the backwater effect of dams creates ponding in the upstream reservoir, producing wetted stream widths and depths greater than downstream of the dam, with the spatial extent of these impacts entirely dependent upon local system channel geometry, channel slope, and height of the dam (Fig 1A) [13, 14, 15]. The combination of backwater ponding effects and partial sediment excavation during high flows in the impoundment are thought to maintain these greater depths and prevent complete sediment infilling of the backwater zone [16]. Second, the combination of some sediment trapping in the impoundment during low flows and the high energy acceleration as flow drops over low-head dams produces scour of the bed and banks, in some cases producing a deep plunge pool and mid-channel bar comprised of coarse scoured material immediately downstream of the low-head dam (Fig 1B). This scour-deposition pattern is caused by transport effective high flows that move sediments through the impoundment and excess energy in the flow that dissipates quickly once the water transits the hydraulic jump at the dam face [16]. If a mid-channel bar does form a short distance downstream from a dam, this may further contribute to channel widening by deflecting flow toward the banks (Fig 1B) [15, 16, 17]. Third, the enhanced flow energy and partial clear water effect immediately downstream of the dam during low and moderate flows induces mobilization of fine fractions of the substrate, producing a coarsening of the substrate below low-head dams, leaving only coarse material (cobble, boulder, bedrock) behind (Fig 1C) [15, 18]. Further downstream of the dam, substrate particle size distributions are assumed to return to the downstream fining pattern in undammed systems (e.g., [19, 20, 21]), but the spatial extent of this adjustment is not well understood (Fig 1C).

Researchers and managers could better understand fragmentation, minimize dam impacts, and conserve aquatic biodiversity if they could quantify the size of geomorphic dam footprints, variation in dam footprint size across dams, and context of this variation. Links between geomorphic and ecological recovery are largely untested but are assumed to be related to one another [22]. Here, we modified and evaluated a method to detect geomorphic changes adjacent to low-head dams, then used this approach to ask three questions. First, we asked if low-head dams have an impact on stream habitat, as measured by wetted width, depth, and substrate size. Relative to this first question, two outcomes are possible: (H_1a_) low-head dams will alter stream habitat upstream and downstream; alternatively (H_1b_) low-head dam impacts will be negligible because these structures are small and recovery is rapid. Second, we asked if low-head dams either (H_2a_) vary in footprint size, as individual dam characteristics may cause differences in the spatial extent of low-head dam impacts, or (H_2b_) did not vary in footprint size. Third, we tested if characteristics of the individual dam (height) and neighboring dams (i.e., number of upstream dams, distance to neighboring dams, and size of neighboring dam) affect footprint size. Our prediction is that the spatial extent of low-head dam impacts (H_3a_) will be affected by dam height and the numbers and locations of neighboring dams. Conversely, (H_3b_) low-head dam impacts may be independent from the numbers, locations, and sizes of neighboring dams. Overall, our research aim is to quantitatively evaluate both the spatial and cumulative extents of low-head dam impacts in order to fill a critical gap in our understanding of anthropogenic controls on fragmented river network ecosystems.

## Materials and Methods

### Study site

**Fig 2:**
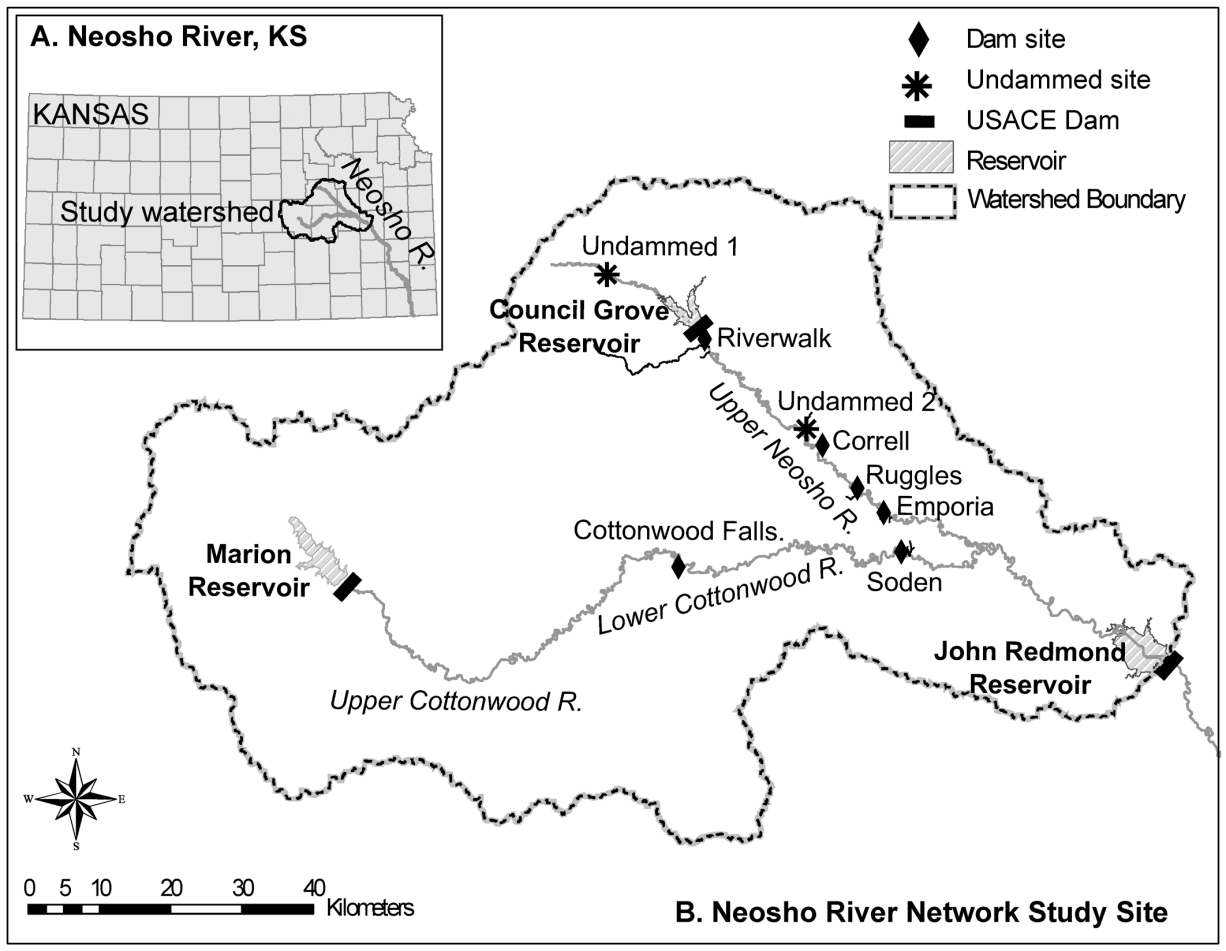
Map of the Upper Neosho River network. Map of our study area in the Upper Neosho river network (A) located in Kansas. Also shown are (B) six dam sites and two undammed reference sites along the Upper Neosho River and Lower Cottonwood Rivers. Major U.S. Army Corps of Engineers (USACE) reservoirs in the study river network are labeled for reference.

The Neosho River is located in the Great Plains, USA and flows southeast for 756 km through Kansas, Arkansas, Missouri, and Oklahoma [23] and drains 32,789 km^2^ of mesic grasslands before joining the Arkansas River in Oklahoma (Fig 2A). The drainage area includes the Flint Hills Upland and Osage Cuestas physiographic regions, which are characterized by gently rolling hills and escarpments [24]. The native vegetation is tallgrass prairie dominated by perennial warm-season grasses. The current land use is primarily agriculture, forest, and rangeland [25]. The study area has a mean annual precipitation of 910 mm [26].

The Upper Neosho River network is located within the 7,000 km^2^ Upper Neosho River basin and includes the 5th order Upper Neosho and 6th order Lower Cottonwood Rivers [27] (Fig 2B). The study area is characterized by an incised, low gradient dendritic drainage pattern with channel slopes of 0.00023 to 0.00057 [28], well-defined steep banks, ranging from 1 to 10 m in height, and channel beds composed of gravel, boulders, and some exposed bedrock. The hydrologic regime is characteristic of the highly variable intercontinental climate, with relatively low mean annual flow and highly variable annual peak flow, typically occurring between April and June (Upper Neosho River: mean annual discharge, 8.7m^3^s^-1^; annual peak flows, 124.6–4,927.3 m^3^s^-1^ (1963–2012; USGS gage 07179730); Lower Cottonwood River: mean annual discharge, 24.4 m^3^s^-1^; annual peak flows, 146.7–26,306.5 m^3^s^-1^ (1963–2012; USGS gage 07182250)[29]. The entire Neosho River basin has high aquatic biodiversity including over 100 species of fish [30, 31] and approximately 35 species of mussels [32]. Many of these aquatic species have life histories adapted to perennial flashy streams (*sensu* [33]).

Wetted width and depth and substrate size were measured at six low-head dams (height 1.2–3 m) and two undammed sites within Upper Neosho River network (Fig 2B). Four low-head dams (Riverwalk, Correll, Ruggles, and Emporia) were located on the Upper Neosho River and two low-head dams (Cottonwood Falls and Soden) were located on the Lower Cottonwood River. The two undammed locations (Undammed-1, 2) were located on the Upper Neosho River, greater than 8 km from the nearest dam. Except for a mill dam downstream of Marion Reservoir on the Upper Cottonwood River, our study included all low-head dams on the Upper Neosho and Lower Cottonwood Rivers between three large U. S. Army Corps of Engineer dams (Marion, Council Grove, John Redmond) (Fig 2B). The six low-head dams were built between the 1860s and 1995 for recreation [34, 35], water supply on the Upper Neosho River [36, 37], and as mills on the Lower Cottonwood River [38, 39] (Table 1). Riverwalk dam is about 20 years old, while the others are all much older (1860–1920). Local municipalities (i.e., Council Grove, Cottonwood Falls, and Emporia) and private landowners gave verbal permission to sample at each of our sampling locations. This field study did not involve any endangered or protected species.

**Table 1:** Date of construction, primary purpose, and drainage area for dams on the Upper Neosho and Lower Cottonwood rivers. Drainage area is cumulative, including parts of the catchment upstream of Council Grove and Marion reservoirs (Fig 2).

River	Dam name	Built	Primary Purpose	Drainage area (km^2^)
**Upper Neosho**	Riverwalk	1995	Recreational	682
	Correll	1920s	Water supply	1,550
	Ruggles	1920s	Water supply	1,600
	Emporia	1890s	Water supply	1,616
**Lower Cottonwood**	Cottonwood Falls	1860s	Mill	3,539
	Soden	1860s	Mill	4,748

### Dam impacts

#### Wetted width and depth

Field survey measurements of wetted width and depth extended 3 km upstream and downstream of dams or until we reached the end of the upstream impoundment (e.g., Riverwalk > 2.2 km), could not obtain landowner permission (Correll downstream > 1 km), or were logistically unable to sample (Emporia upstream). Wetted width, or the width of the water surface at the time of observation, was measured using a laser range finder (Nikon Archers Choice with < 1 m accuracy, range 3–200 m). Bankfull widths and depths were not consistently measurable in the field because of the scale of the river channel and challenges associated with physically exiting the channel (dense vegetation, vertical high banks) during surveys. To ensure comparable flows, wetted widths and depths for all sites were sampled within a five day period near baseflow conditions, according to the baseflow index [40], and USGS period of record for gages on the Neosho (baseflow: 0.57 m^3^s^-1^; field survey: 0.65m^3^s^-1^; USGS gage 07179730) and Cottonwood Rivers (baseflow: 2.5m^3^s^-1^; field survey: 1.4 m^3^s^-1^; USGS gage 07182250).

Depth was measured at five regularly-spaced points along each transect with a meter stick (< 1 m depth) or a depth finder (Lowrance X-4) attached to a kayak (> 1 m depth). Wetted width and depth upstream and downstream of each dam were compared at transects spaced every 200 m for the first kilometer and 500 m thereafter, starting at 200 m (*N* = 9 transects). The difference in wetted width and depth between upstream and downstream reaches was evaluated using a non-parametric Wilcoxon rank sum statistic (W) [41]. For all statistics on which we ran multiple tests, we calculated a Bonferroni family-wise error rate starting with α = 0.05 divided by the number of tests. Because the number of tests varies, for all subsequent statistics, we note the corrected Bonferroni critical alpha that we used to determine significance. For example to compare upstream and downstream wetted widths, we divided our original alpha (0.05) by five dam comparisons to create a new critical alpha of 0.01 on which significance was based.

#### Quantifying the geomorphic dam footprint

**Fig 3:**
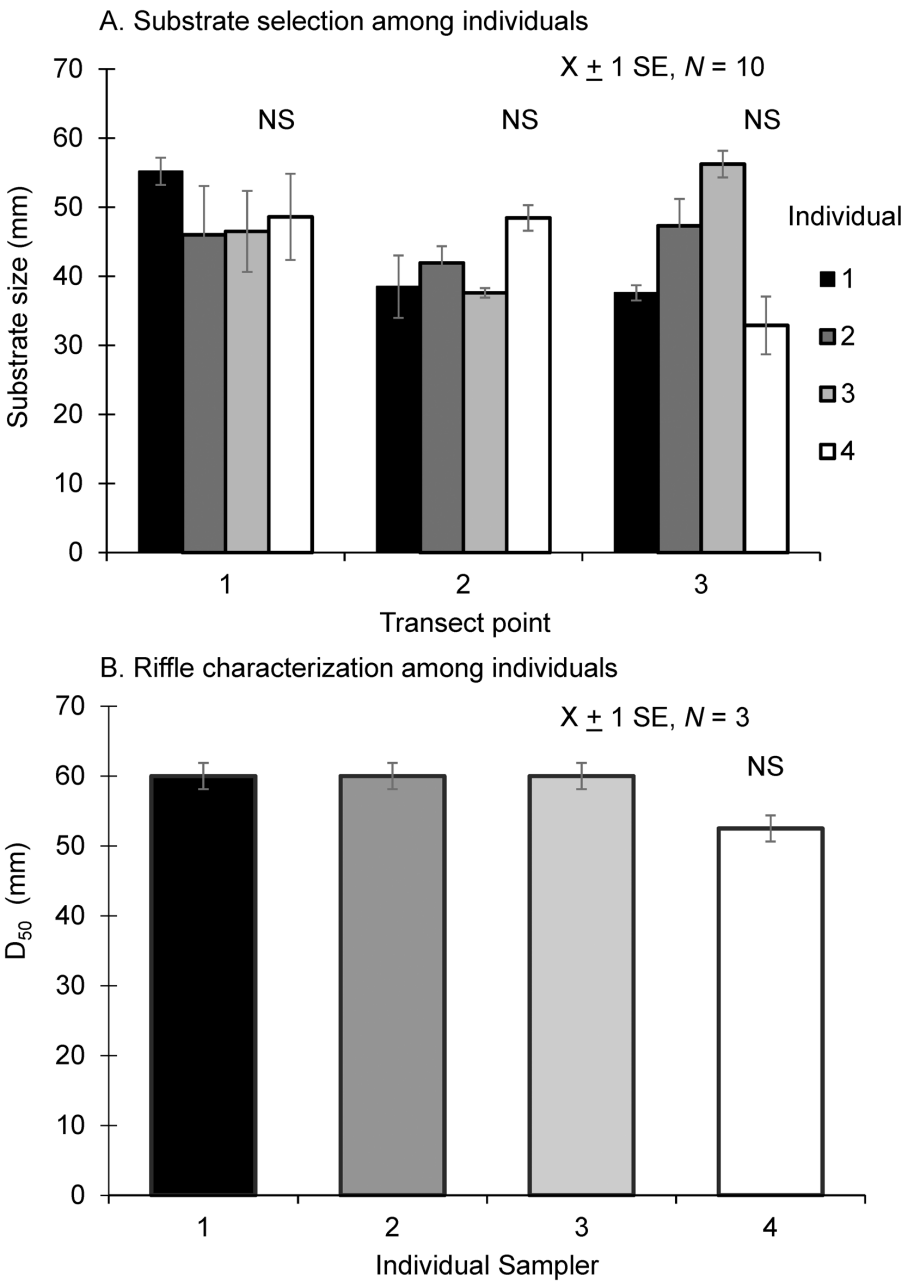
Evaluation of Substrate Selection among Individuals. Comparison of (A) mean substrate size picked by four individuals at three randomly chosen points along a transect with ten replicates for each point and (B) average D_50_ of three Wolman pebble counts of one riffle. NS indicates no significant difference between individuals. Statistics are the result of a Kruskal-Wallis test of sampler effect using (A) a Bonferroni corrected α = 0.016 (0.05 divided by 3 (for each location)) (B) α = 0.05.

Substrate sizes were characterized in the field following a careful evaluation of potential individual sampler bias in substrate size selection (*sensu* [42]). First, we tested if substrate size selection by four different individuals was more variable than repeated selection of substrate by a single individual (10 replicates) at the same location along a transect using a randomized block design. Substrate was measured along the intermediate axis with a gravel template (gravelometer, 2–362 mm). All individual samplers selected similar-sized substrates (+/- one size class of the gravelometer) at three predetermined locations along a transect (location 1 (*chi-sq* = 0.72, *df* = 3, *P* = 0.87), location 2 (*chi-sq* = 1.31, *df* = 3, *P* = 0.73), location 3 (*chi-sq* = 7.25, *df* = 3, *P* = 0.06); Kruskal-Wallis test, α = 0.016 with Bonferroni correction; Fig 3A). Second, we tested if D_50_ (the median substrate size, e.g., [19]) from a standard Wolman pebble count on a riffle [43] was more variable among individuals than repeated counts by a single individual (three replicates) for the same riffle. In the second evaluation of individual sampler effects, D_50_ was not different among the four individuals (Kruskal-Wallis test: *chi-sq* = 6.55, df = 3, *P* = 0.09, α = 0.05; Fig 3B).

Because our standard protocols controlled individual sampler effects satisfactorily, next, we used a standard Wolman pebble count [43] to determine substrate size composition three km downstream of six dams and at two undammed sites (a distance approximately 100 times wetted width). We sampled at standard geomorphic units (i.e., riffles) to prevent local sorting of sediment from confounding longitudinal patterns in substrate sizes [44]. All riffles downstream of each dam were sampled the same day and reference sites were sampled within 24 h to ensure comparable flow conditions. When a large distance separated riffles (>1 km), we measured channel depths every 200 m to ensure that no riffle was missed. The end of the downstream dam footprint was defined as the downstream location at which median (D_50_) substrate size reached a baseline determined by the undammed sites, or the minimum value for (D_50_) on its river (Upper Neosho or Lower Cottonwood). In general, substrate size gradually decreases as one goes downstream in a river because of some combination of hydraulic sorting (selective entrainment of finer sediments), abrasion and weathering [45]. The general trend is punctuated by larger mean substrate size downstream of tributaries and barriers in gravel-bed rivers (e.g., [19, 45]). We feel confident in using a baseline substrate size to determine the extent of dam footprints because (a) the Neosho River network is a low gradient system, (b) the variation in channel slope and discharge within the study area was negligible, (c) the major change in channel slope occurs downstream of John Redmond Reservoir [28], and (d) none of the sites were located downstream of the Upper Neosho and Lower Cottonwood confluence.

We plotted cumulative distribution curves of the substrate for each riffle to examine the entire substrate profile in addition to the median substrate size to support our results. A two-sample Kolmogorov-Smirnov test was used to determine if the cumulative distribution curves from the first riffle downstream of a dam was statistically different from a riffle outside the downstream dam footprint [41].

We supplemented field surveys with aerial imagery [46] to help locate the extent of impoundments and channel widening. Channel widths and visibility of riffles are affected by flow. The aerial images were taken at or below baseflow conditions according to the baseflow index [40], and USGS period of record for gages on the Neosho (baseflow: 0.57 m^3^s^-1^; aerial image: 0.54 m^3^s^-1^; USGS gage 07179730) and Cottonwood Rivers (baseflow: 2.5 m^3^s^-1^, aerial image: 0.79 m^3^s^-1^; USGS gage 07182250). Upstream, we quantified the extent of the upstream dam impoundment by identifying the first gravel bar or riffle upstream of the dam, and then ground-truthed this location in the field. The end of the upstream dam footprint was defined as the location of the first riffle or gravel bar above the dam. Downstream of dams, we measured the longitudinal distance of channel widening from the dam spillway to the point where channel width from the aerial image [46] returned to the average wetted width of that site calculated from field surveys.

#### Patterns of variation in dam footprints

We examined patterns of variation in geomorphic footprints across dams in three ways. First, we compared size of dam footprints across individual dams within the Upper Neosho river network. Second, to understand variation in footprints across dams, we tested if dam height, number of upstream dams, distance to nearest upstream dam, size of the nearest upstream dam, and drainage area were related to the size of the dam footprint (downstream, upstream, and total) using univariate regression (*N* = 6 dams) [41]. The total footprint was the sum of the upstream and downstream substrate footprints. Each dam’s position in the river network, relative to other dams, was measured along the river flowline [47, 48]. Variations in substrate footprints were analyzed and expressed in multiples of mean wetted width from field survey measurements taken on the Upper Neosho and Lower Cottonwood rivers, respectively (Table 2). Regression analyses with kilometers as the measurement unit yielded similar results. By expressing our findings in multiples of wetted widths, our results can be easily compared across river networks.

**Table 2:** Channel widening, downstream, upstream, and total footprint for each dam. Downstream footprints were determined by measuring the distribution of median substrate size (D_50_) from riffles downstream of dam (see Figs 6 and 8). Extent of channel widening, and upstream footprints were determined using aerial photography. Footprints are expressed in terms of multiples of mean wetted width, with kilometers in parentheses. The mean wetted width used in calculations was 0.022 km for the Neosho River and 0.035 km for the Cottonwood River.

River	Site	Channel widening (No. widths (km))	Upstream footprint (No. widths (km))	Downstream footprint (No. widths (km))	Total footprint (No. widths (km))
**Upper Neosho**	Riverwalk	2 (0.05)	100 (2.2)	59 (1.3)	160 (3.5)
	Correll	11 (0.25)	300 (6.6)	55 (1.2)	355 (7.8)
	Ruggles	10 (0.21)	295 (6.5)	55 (1.2)	350 (7.7)
	Emporia	9 (0.2)	127 (2.8)	9 (0.2)	136 (3)
**Lower Cottonwood**	Cottonwood Falls	6 (0.22)	391 (13.7)	46 (1.6)	437 (15.3)
	Soden	6 (0.21)	245 (8.6)	40 (1.4)	286 (10)
**Overall**	Mean	7 (0.19)	243 (6.7)	44 (1.2)	287 (7.9)
	Total	44 (1.14)	1458 (40.4)	264 (6.9)	1,724 (47.3)
	Range	2, 11 (0.05, 0.25)	100, 391 (2.2, 13.7)	9, 59 (0.2, 1.6)	136, 437 (3.0, 15.3)

* footprint is estimated based on available data

## Results

### Wetted width and depth

**Fig 4:**
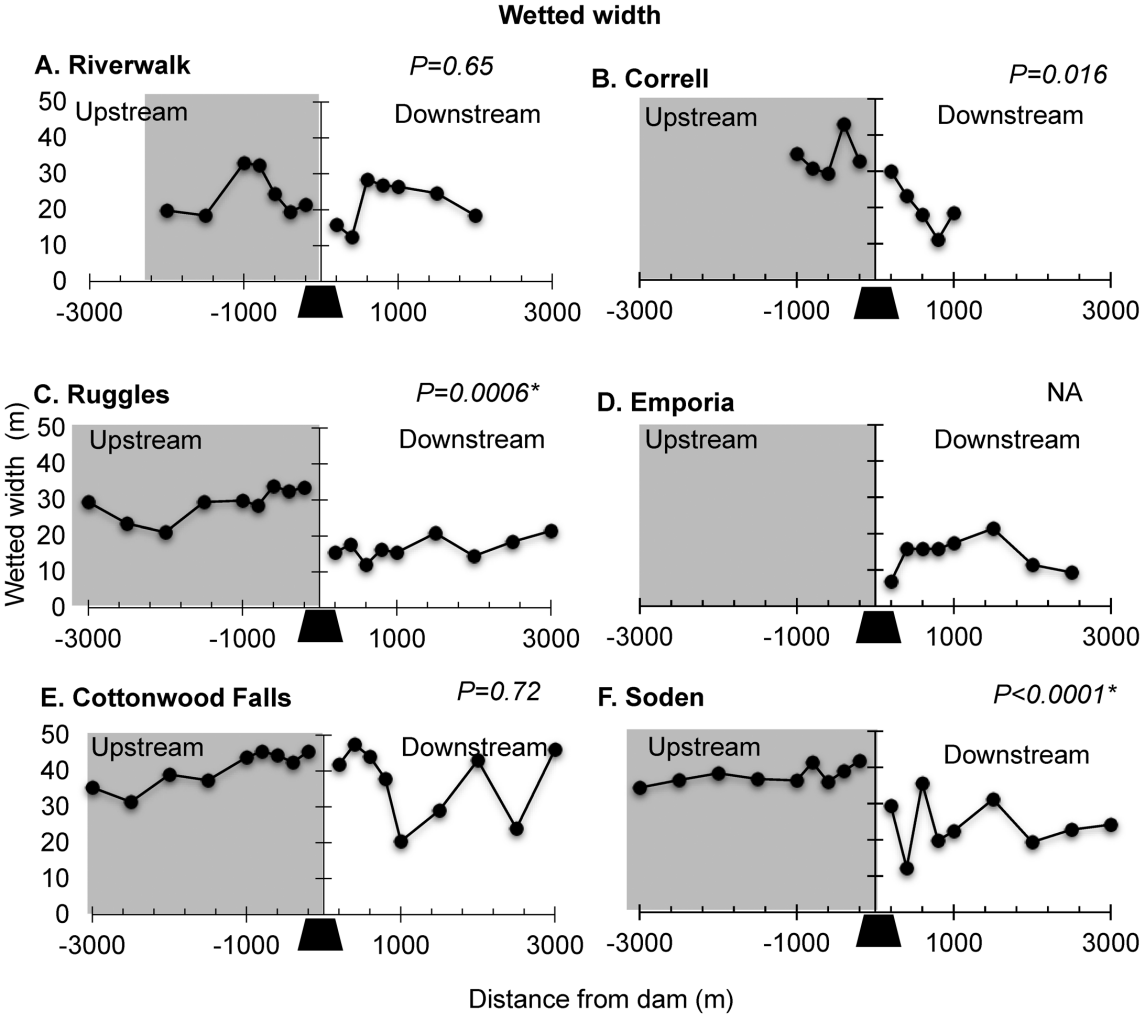
Wetted Width. Longitudinal profiles of wetted width for the six dams in the study reach showing upstream (left) and downstream (right) samples for our six study dams (black trapezoid); (A) Riverwalk, (B) Correll, (C) Ruggles, (D) Emporia, (E) Cottonwood Falls, and (F) Soden. Y axis arrows indicate mean wetted width. Small arrows in panel indicate footprint based on substrate size profiles see section on substrate size for details. *P-values* from Wilcoxon rank sum test for mean differences between upstream and downstream transects are shown. Asterisk indicates significance with Bonferroni corrected α = 0.01 (0.05 divided by five dams).

Wetted width was not consistently different between upstream impoundments (range 20–45 m) and downstream of dams (range 7–47 m). Although mean wetted width was greater upstream at all sites (Fig 4), upstream reaches were significantly wider than downstream reaches at two of the five dams for which we had upstream and downstream data (Ruggles dam (*P* = 0.0006, Fig 4C); Soden dam (*P*<0.0001, Fig 4F)). Correll was marginally different using the Bonferroni family wise error rate (*P* = 0.016, Fig 4B). Riverwalk and Cottonwood Falls were not statistically different (Fig 4A and Fig 4E).

**Fig 5:**
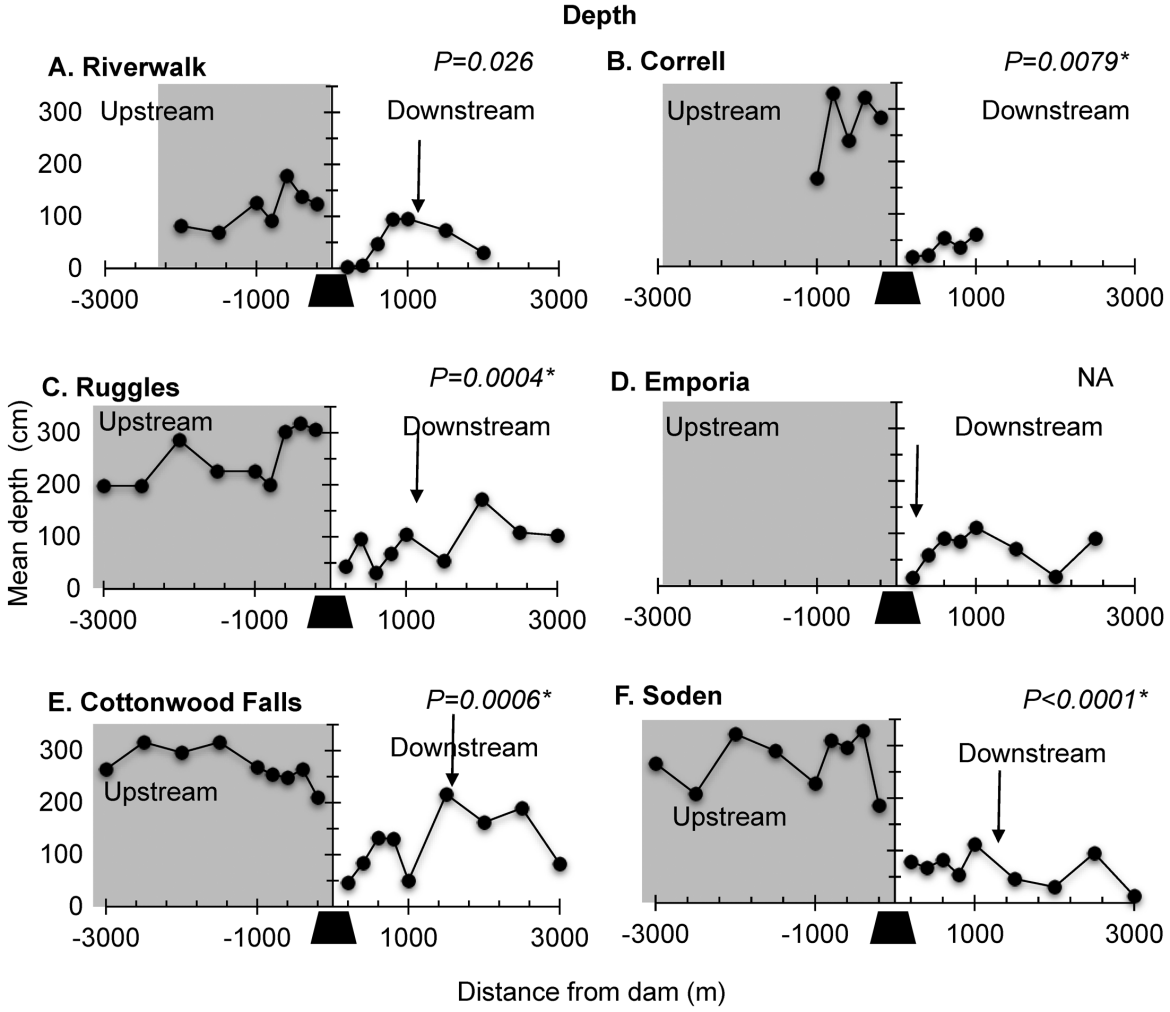
Depth. Longitudinal profiles of depth for the six dams in the study reach showing upstream (left) and downstream (right) samples for our six study dams (black trapezoid). (A) Riverwalk, (B) Correll, (C) Ruggles, (D) Emporia, (E) Cottonwood Falls, and (F) Soden. Y axis arrows indicate mean depth. Small arrows in panel indicate footprint based on substrate size profiles (see section on substrate size for details). *P-values* from Wilcoxon rank sum test for mean differences between upstream and downstream transects are shown. Asterisk indicates significance with Bonferroni corrected α = 0.01 (0.05 divided by five dams).

Depth also was not consistently different between upstream impoundments (82–330 cm) and downstream (range 3–216 cm) of dams. At all sites, mean depths upstream of dams were generally greater than mean depths downstream of dams, but significantly different at four of the five dams for which we had upstream and downstream data (Correll, *P* = 0.0079, Fig 5B; Ruggles, *P*<0.004; Fig 5C; Cottonwood Falls, *P*<0.0006; Fig 5E; Soden, *P*<0.0001; Fig 5F)). Riverwalk was not significantly different upstream compared to downstream of the dam (Fig 5A). Neither width nor depth revealed consistent longitudinal trends with increasing distance from the dam.

### Substrate Size

**Fig 6:**
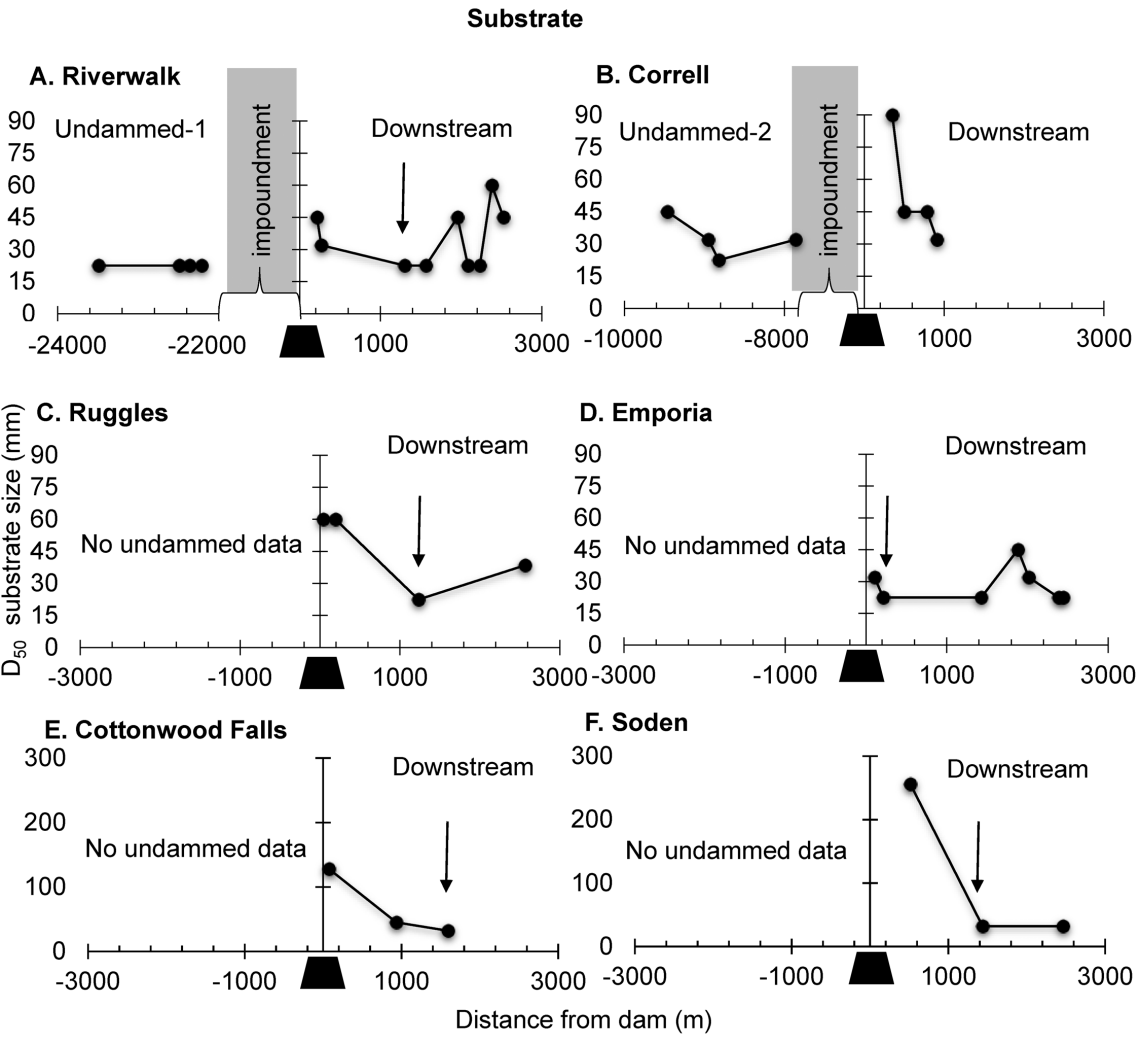
Substrate size. Longitudinal profiles of substrate size for the six dams in the study reach showing upstream undammed sites as available (left) and downstream (right) samples (black trapezoid). Each point represents a riffle’s location in relation to its distance from the dam. The end of the dam footprint is indicated by an arrow, where applicable, and was considered to be where riffles reached a baseline determined by the undammed sites, or the minimum value for (D_50_) on its river (Upper Neosho or Lower Cottonwood). (A) Riverwalk, (B) Correll, (C) Ruggles, (D) Emporia, (E) Cottonwood Falls, and (F) Soden. The longitudinal profile for the two reference sites, Undammed-1 and Undammed-2 are plotted in the upstream panel for their corresponding dam sites, Riverwalk and Correll, respectively.

Substrate size consistently detected a longitudinal recovery from dam effects. Riffles at the undammed reference sites (Undammed-1 (Fig 6A); Undammed-2 (Fig 6B)) had a D_50_ from 22.5 to 45 mm (Fig 6A and 6B). Immediately downstream of all dams, D_50_ was larger than at reference sites (Fig 6A–Fig 6F), decreased below all dams to 22.5 mm at 0.21–1.2 km downstream of Upper Neosho River dams (Fig 6A, Fig 6C and Fig 6D), and to 32 mm at 1.4 and 1.6 km downstream of both Cottonwood River dams (Fig 6E and Fig 6F). Substrate size increased downstream of tributary junctions (Fig 6A and Fig 6D).

**Fig 7:**
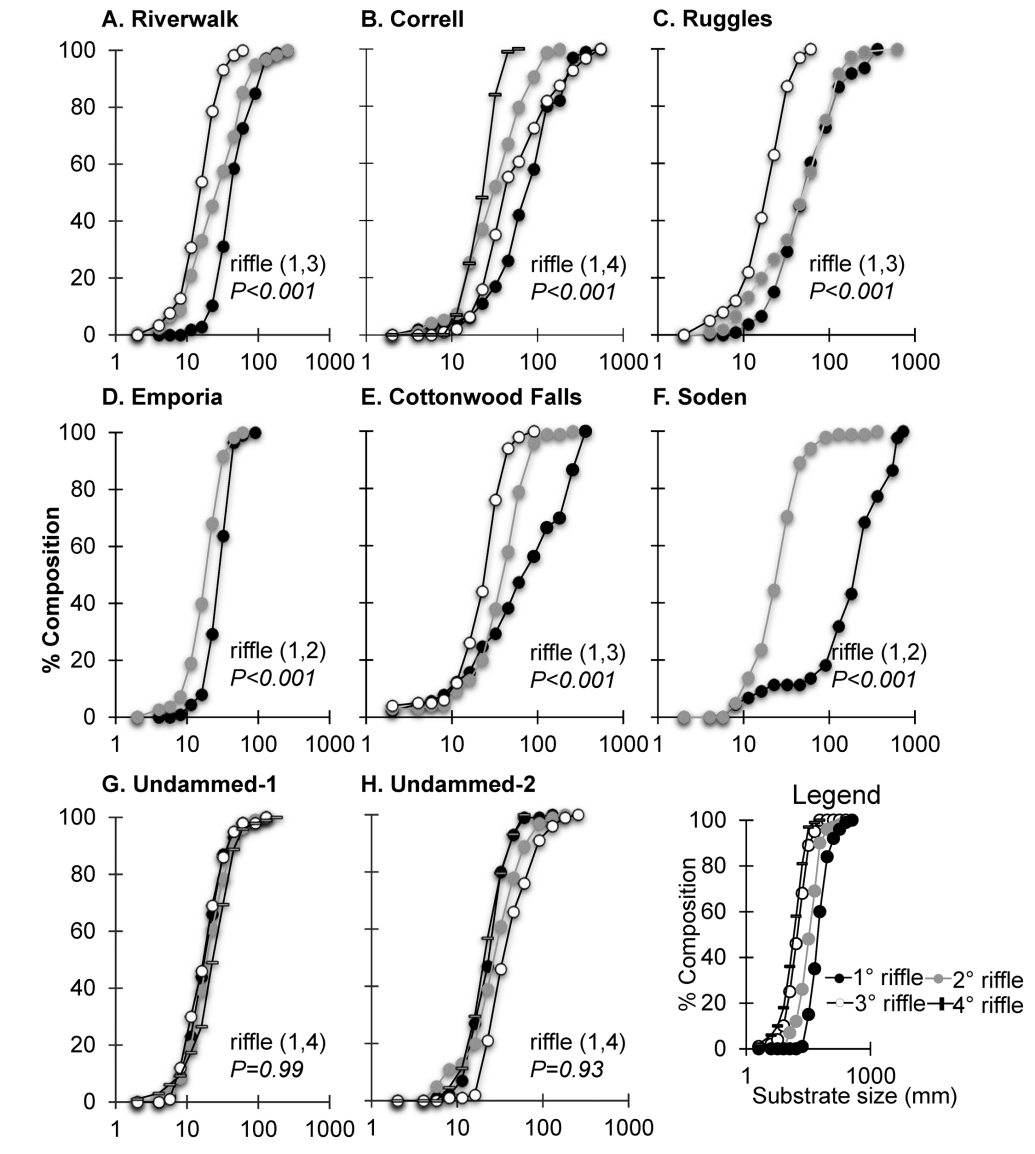
Cumulative Distribution Curves. Upper panels display substrate size composition changes with increasing distance from dams for (A) Riverwalk, (B) Correll, (C) Ruggles, (D) Emporia, (E) Cottonwood Falls, (F) Soden, and undammed sites (G) Undammed-1 and (H) Undammed-2. Consecutive riffles below the dam are displayed until median substrate size (D_50_) returned to 22.5 mm for Neosho River or 32 mm for Cottonwood River. For comparison, lower panels display substrate size compositions for riffles at reference sites located away from dams where distributions remained similar. Note: reference sites with riffles were not available for four of the six dams. *P-values* in the figures are based on Kolmogorov-Smirnov test of the distribution curves comparing the two riffles indicated in parentheses. See panel I for the legend—1° riffle indicates the first riffle downstream of a dam, 2° riffle indicates the second riffle downstream of a dam, 3° riffle indicates the third riffle downstream of a dam.

Cumulative distribution curves of riffles displayed fining in which riffles closest to the dam (1^st^ riffle) had the largest substrate sizes (Fig 7A–Fig 7F). Undammed sites did not display this fining from an upstream to downstream direction (Fig 7G and Fig 7H). Riffles farthest from the dam had higher percentages of small substrates (unless a tributary joined the mainstem). The substrate distribution curves of the riffles closest to the dam were significantly different from those of riffles outside the dam footprint (Fig 7A–Fig 7F). As with D_50_, patterns of D_16_ and D_84_ substrate size fractions were largest immediately downstream of dams (Upper Neosho R (D_16:_ 22.5–32 mm; D_84:_ 45–256 mm); Lower Cottonwood R (D_16_: 22.5–122; D_84_: 362–618)). Undammed sites had consistent and overlapping substrate size distributions (Fig 7G and Fig 7H).

### Geomorphic low-head dam footprint

**Fig 8:**
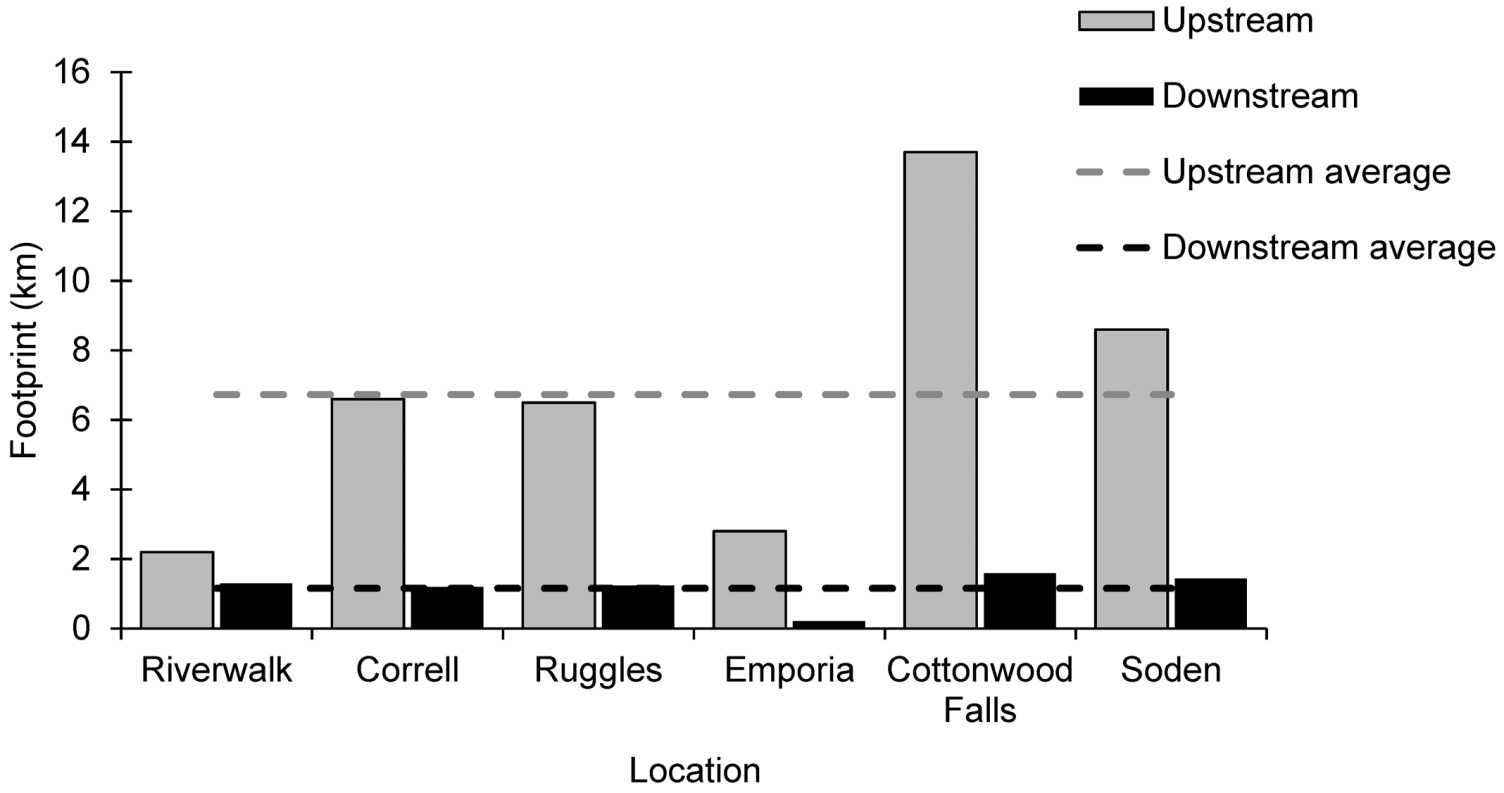
Low-head Dam Footprints. Downstream (black bars) and upstream (gray bars) footprints of six dam sites and the average downstream and upstream footprints in the Neosho River network. The dashed lines are averages.

The spatial extent of the downstream dam substrate footprint ranged from 0.2 to 1.6 km (9 to 59 wetted widths) with a mean of 1.2 km or 44 wetted widths (Fig 8 and Table 2). The downstream geomorphic footprints of all low-head dams were relatively similar (40–59 wetted widths) with the exception of Emporia’s smaller (9 wetted widths) downstream footprint. At all dam sites, downstream impact of channel widening (0.05–0.25 km; 2–11 wetted widths) extended a shorter distance than the downstream substrate footprint (t = -5.82, df = 5, *P* = 0.0021) (Table 2).

The upstream dam footprints extended 100–391 wetted widths (2.2–13.7 km) with a mean of 243 wetted widths (6.7 km) (Fig 8 and Table 2). Upstream footprints were larger than downstream footprints at all sites (t = -4.33, df = 5, *P = 0*.*0067*). Total dam footprints (upstream plus downstream) averaged 287 wetted widths (7.9 km) and ranged from 136 to 437 wetted widths (3.0–15.3 km) (Table 2). A total of 47.3 km (1,724 wetted widths) of stream habitat on the mainstem in the Upper Neosho River network (17%) was altered by these six intact low-head dams (Table 2).

### Patterns of Variation in Dam Footprints

**Fig 9:**
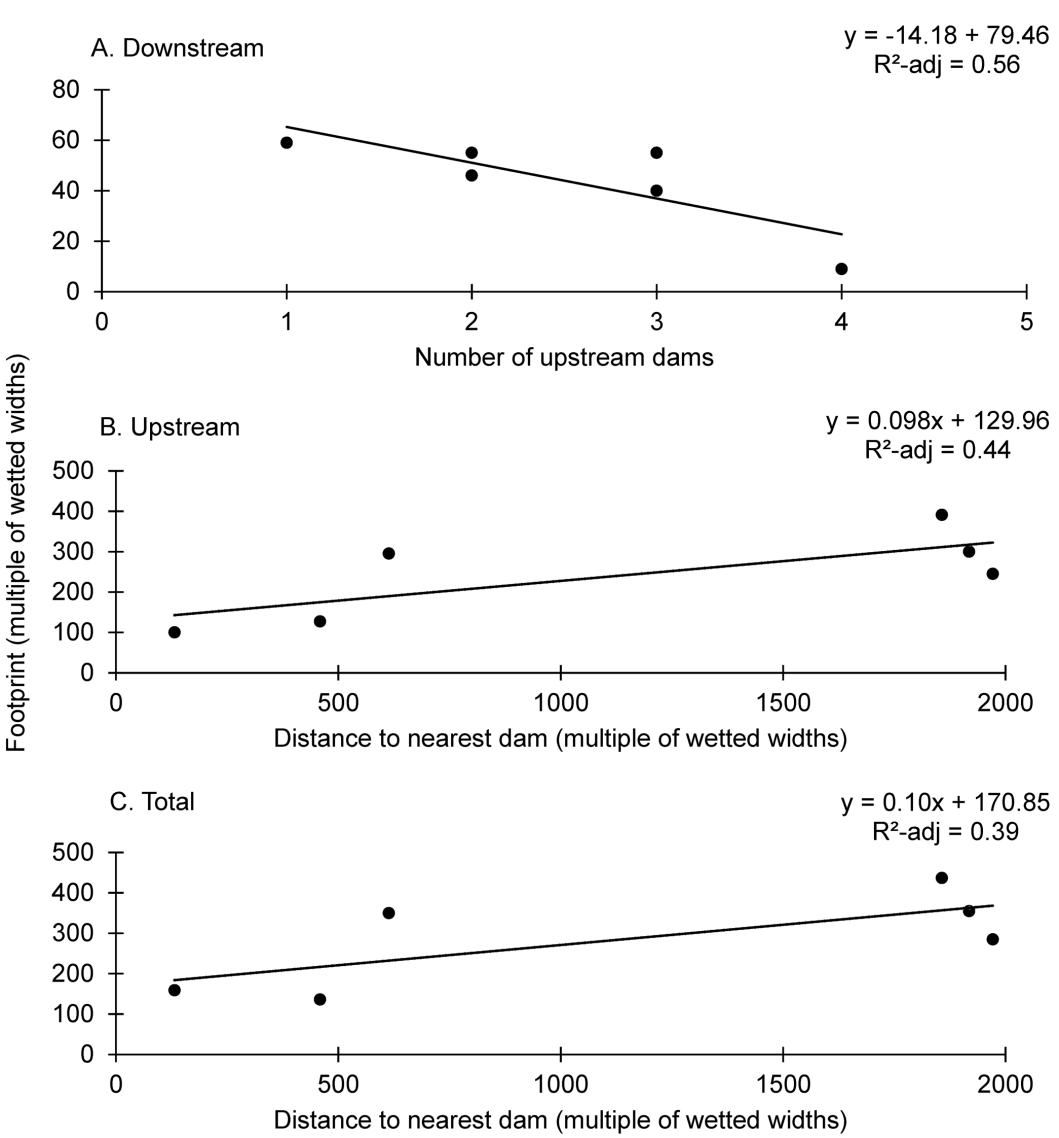
Regression for Environmental Correlates of Dam Footprints. Univariate regressions for environmental correlates of (A) downstream, (B) upstream, and (C) total dam footprints expressed in terms of multiples of wetted widths. The relationship shown corresponds to the top model amongst competing univariate regressions testing dam height, distance to nearest upstream dam, number of upstream dams, and height of nearest upstream dam. The corresponding equation and correlation (adjusted R-sq) between the dam footprint and the corresponding explanatory variable are indicated in each panel.

Number of upstream dams was inversely related to downstream footprint size and explained 56% of variation in the downstream footprint (Y = -14.18 (SE ± 5.25) X + 79.46 (SE ± 14.05); *P* = 0.054, adjusted *R* ^*2*^ = 0.56; Fig 9A). On the Lower Cottonwood River, Cottonwood Falls and Soden had two and three upstream dams, respectively. On the Upper Neosho River, the most upstream low-head dam, Riverwalk, had one upstream dam, namely Council Grove Reservoir, and the most downstream dam, Emporia, had four upstream dams (Fig 2 and Table 3). The other four variables (dam height, distance to the nearest dam, height of the nearest upstream dam, and drainage area) were not present in top models (ΔAIC > 2).

**Table 3:** Dam size and river network context relative to other dams in the river network (number of upstream dams, distance to nearest upstream dam, height of 1^st^ upstream dam, and drainage area). Input variables for univariate regressions were standardized to multiples of mean width, except for drainage area, for the Upper Neosho and Lower Cottonwood rivers, respectively.

River	Dam name	Dam height (No. widths (km))	No. upstream dams	Distance to nearest upstream dam (No. widths (km))	Height of 1st upstream dam (No. widths (km))	Drainage area (km^2^)
**Upper Neosho**	Riverwalk	0.05 (1.2)	1	131.82 (2.9)	1.33 (29.3)	682
	Correll	0.10 (2.3)	2	1,918.18 (42.2)	0.05 (1.2)	1,550
	Ruggles	0.11 (2.4)	3	613.64 (13.5)	0.10 (2.3)	1,600
	Emporia	0.14 (3)	4	459.09 (10.1)	0.11 (2.4)	1,616
**Lower Cottonwood**	Cottonwood Falls	0.09 (3)	2	1857.14 (65.0)	0.09 (3)	3,539
	Soden	0.09 (3)	3	1971.43 (69.0)	0.09 (3)	4,748

Distance to the nearest dam was positively related to upstream (Y = 0.098 (SE ± 0.044) X + 129.92 (SE ± 61.45); *P* = 0.10; adjusted R^2^ = 0.44; Fig 9B) and total footprint sizes (Y = 0.10 (SE ± 0.049) X + 170.8 (SE ± 68.37); *P* = 0.11; adjusted R^2^ = 0.39; Fig 9C)). For Riverwalk, a large USACE dam (29.3 m high) was only 2.9 km upstream. For Emporia and Ruggles, the upstream low-head dams were close (10.1–13.5 km). Whereas for Correll, the nearest dam was about 42 km away, and for Cottonwood Falls and Soden the nearest upstream dam was located at a much greater distance (65–69 km). The variables dam height, number of upstream dams, and drainage area were not present in top models for upstream or total footprint (ΔAIC > 2).

## Discussion

Here we quantified the geomorphic dam footprints at six low-head dams. Field surveys of substrate size distributions documented consistently larger substrate size immediately below dams, a consistent longitudinal decrease in substrate size downstream of dams, and a consistent return to baseline (i.e., analogous to undammed sites) substrate size along a longitudinal recovery at each of our study sites. Past geomorphic research [13, 15, 18, 19], showed nonsignificant coarsening (i.e., increased size) of substrate occurs immediately downstream of low-head dams. [15, 18]. In contrast, our results showed consistently significant larger substrate sizes downstream of dams compared to riffles outside the footprint. Methodological differences, such as the use of regularly spaced transects (e.g., [15, 18]) as opposed to repeating geomorphic units (this study), may have confounded dam effects with those related to local sorting of sediment [44].

Substrate is an ecologically valuable indicator of the dam footprint because substrate can affect the structure of invertebrate communities and survival and reproductive success of fishes (e.g., [49, 50]). Measuring the dam footprint explicitly measures habitat characteristics (e.g., substrate, hydraulic habitat regime) on which aquatic biodiversity depends (e.g., [10]). Aquatic organisms are adapted to the lateral and longitudinal connectivity of geomorphic, hydrologic, and ecological processes in lotic ecosystems [1–2, 51]. The relationship between organisms and their habitat makes the spatial frame of dam induced habitat changes (single or cumulative) relevant to patterns in biodiversity. Maintaining biodiversity is a priority because biodiversity can affect ecosystem function [52, 53], act as a useful indicator of anthropogenic stress, and provide a foundation for effective conservation practices. Without knowing where the geomorphic dam footprint ends, downstream dam impacts on biodiversity and individual variability across dams will be difficult to assess, interpret, and generalize.

Many of our results, though not all, were consistent with previous findings regarding the geomorphic impact of low-head dams. As reported in other studies, we found that upstream dam impoundments were variable and not consistently statistically deeper or wider than downstream of low-head dams (e.g., [14, 15, 18]). In the Upper Neosho River network, differences in upstream widths were likely due to the passive inundation of the channel caused by the dam, whereas differences in upstream depths were likely due to impoundment-specific bedload transport regimes during high flows that determined scour of accumulated sediments (*sensu* [16]). Changes in width and depth around dams depend on many local factors including underlying geology, channel confinement, channel slope, and height of dam relative to bank height, so they will vary within and between river systems. The lack of clear and consistent longitudinal patterns in the downstream direction of wetted width and depth channel geometry parameters reduced the usefulness of wetted width and depth as indicators of low-head dam footprint lengths. Although channel widening has been frequently documented [15, 17, 18], the spatial extent of channel widening did not correlate well with our other measured geomorphic parameter—substrate size. For example, at Ruggles Dam, channel widening extended 9 wetted widths (0.2 km) downstream of the dam, while substrate size did not return to the 22.5 mm until 55 wetted widths (1.2 km) downstream. The dam-related process of altered sediment supply-transport capacity leading to channel adjustments is understood (e.g., [54]) but, to our knowledge, ours is the first study to evaluate the longitudinal pattern of substrate size distribution with repeating geomorphic units at low-head dams.

Dams alter and fragment large sections of river networks, especially when multiple low-head dams are present within a network [55]. Footprint size was a useful response. In addition, footprint size, expressed as multiples of wetted width, may explain across-site and across-system variation and could provide a metric useful to others working in different streams. Our spatially extensive data set revealed that the total spatial extent of low-head dam footprints can be quite substantial (7.9 km or 287 multiples of wetted width per dam on average). The average downstream component of this geomorphic dam footprint for the six dams in the Upper Neosho River network averaged 55 wetted widths (1.2 km). Although smaller than the reported spatial impact downstream of large dams (> 7.6 m high), which may extend up to hundreds of kilometers (e.g., [56]), this downstream geomorphic footprint for low-head dams is more significant than what has been predicted from channel widening or assumed previously (< 100 m (e.g., [7]); < 500 m (e.g., [6])). When examined cumulatively from a network perspective, 47.3 km of the Upper Neosho River network, or about 17%, was physically altered by low-head dams, resulting in a substantial overall impact of multiple low-head dams along the mainstem of the river network.

The individual and cumulative extents of low-head dam footprints are affected by geographic location/geology, position in a drainage, position relative to other dams higher in a drainage, and sediment supply [13]. In the Upper Neosho river network, variables regarding position relative to other dams were better predictors of the size of downstream and upstream dam footprints than their position in the drainage (represented by drainage area upstream of dams). The downstream dam footprint was negatively correlated with the number of dams upstream (i.e., more upstream dams often resulted in a smaller footprint). The number of upstream dams is important because sediment storage by upstream dams can limit sediment delivery to downstream dams [13]. For example, Emporia dam had the smallest downstream footprint at 9 wetted widths (0.21 km), and was located below four dams in the river network. The dams above Emporia dam (Correll, Ruggles), were close together in the study site (13 and 10 km apart). Sediment starvation from their impoundments, and limited recovery distance for the input of coarse substrates from tributaries likely exacerbated substrate fining at Emporia. Thus, substrate below Emporia dam returned to baseline sizes quickly, resulting in a smaller footprint, because of sediment starvation due to sediment storage in the impoundments of dams upstream. In this context, multiple dams decreased geomorphic footprint size, and the number and proximity of neighboring dams influenced the extent of impact. Although the age of structures could influence dam impacts because of cracks or partial breaches [13], in our study, age differences did not appear to be a factor because all of the concrete structures remained intact. The upstream dam footprint was positively correlated with distance to the nearest dam. Although the mechanism behind this pattern requires further investigation, decreases in water surface slope imposed by neighboring dams may increase the size of upstream dam footprints [54, 57] because the extent of impoundments depends on a ratio of dam height to channel slope [13]. More study is needed to confirm the underlying processes of patterns in upstream and downstream footprints, but our results show that the context (e.g., proximity to and number of neighboring dams) needs to be included in any future evaluations of low-head dam impacts, in addition to the individual characteristics of each dam.

The next steps for testing low-head dam effects are critical for research and conservation but also challenging. More samples of dam footprints across river networks are necessary to draw generalities about the size of low-head dam impacts. However, increasing sample sizes for testing low-head dam effects, especially in a network context, will not be easy because incorporating dams from other river networks to ensure a desirable sample size will also add additional sources of variation. Thus, increasing sample size of comparable dams will always be a problem. Although we had a limited number of samples (i.e., six dams), they were all within the same river network and had comparable geographic/geologic properties. Furthermore, our sample size exceeded other low-head dam studies which typically have sampled at only one or two dams [13].

Dam size, appropriate undammed references, and a limited understanding of the link between hydrologic, geomorphic and ecological recovery present future research and management challenges for understanding the effects of low-head dams. Research that has shaped our thinking about dam impact has largely been undertaken on large dams with clear impacts [12]. Low-head dams may be relatively small but they are more numerous [9]; by number, they may dominate the fragmentation problem (e.g., [58]). Thus, quantifying cumulative effects and the spatial context of low-head dams within river networks will require a fundamentally different approach to studying low-head dams than the isolated approach that has been applied to larger dams. This neighborhood context is particularly unique to low-head dams because their size and abundance make their effects different from large dams. Finally, we assume geomorphic recovery and ecological recovery are linked [22], but these complex and highly variable relationships are rarely tested and require additional interdisciplinary research.

A quantitative measure of dam footprint facilitates testing how dams interface with a wide range of ecological concepts (e.g., thresholds, disturbance, and edge-effects). For example, dams or their footprints may create habitat edges producing behavioral responses that may help explain observed phenomenon in species distributions (*sensu* [59]). Differences in width and depth between upstream and downstream could function as breakpoints (*sensu* [60]) where dams separate habitats (lentic upstream vs lotic downstream) which are important determinants of macroinvertebrate (e.g., [61]), mussel (e.g., [62]), and fish (e.g., [5]) distributions. However, comparable breakpoint research that relates geomorphic (e.g., habitat structure) and ecological (e.g., organismal) patterns of low-head dam impacts has not been undertaken in geomorphology [13] and rarely in stream ecology.

Understanding dam footprints also provides a foundation for more effective conservation planning (e.g., establishing baseline data before dam removals or quantifying recovery trajectories) [63]. It is imperative that aquatic scientists use a holistic and interdisciplinary perspective when studying and managing low-head dams. Interest in and literature about low-head dams is growing because many dams are reaching the end of their lifespan and being considered for removal for safety and other reasons [64]. A call exists for the formal classification of all dams [12] because limited guidance exists about how to manage dams. Our novel approach to quantifying spatial extent of dam footprints to detect low-head dam effects and individual variability across dams can guide ecological research, restoration, and environmental evaluation related to anthropogenic impacts of fragmentation.

In summary, our standardized and generalizable methodology quantified changes in riffle substrate size and channel geometry, documented the spatial extent of these impacts and explored their variability within the network context of the Upper Neosho river network. This research approach can easily be applied elsewhere to consistently detect geomorphic dam footprints and longitudinal recovery trajectories and to better inform ecologists and environmental professionals as they seek knowledge to guide management of low-dams within riverscapes.

## Supporting Information

S1 Table Literature Review of Geomorphology and Low-head Dams. (DOCX)
