# Supplementary material for: How Big of an Effect Do Small Dams Have? Using Geomorphological Footprints to Quantify Spatial Impact of Low-Head Dams and Identify Patterns of Across-Dam Variation
Source: PLoS One. 2015 Nov 5;10(11):e0141210. doi: 10.1371/journal.pone.0141210 (PMC4634923; doi:10.1371/journal.pone.0141210)
Supplement: S1 Table — (DOCX) [file pone.0141210.s001.docx]

| **Categorization** | **References** |
| --- | --- |
| **Flow model** | (1) Abad JD, Rhoads BL, Güneralp I, García MH. Flow Structure at Different Stages in a Meander-Bend with Bendway Weirs. J Hydraul Eng 2008;134(8):1052-1063. |
| **Sediment yield** | (2) Barendregt RW, Ongley ED. Piping in the Milk River Canyon, southeastern Alberta - a contemporary dryland geomorphic process. IAHS Publication 1977;(122):233-243. |
| **Not about dam** | (3) Bhagat VS, Sonawane KR. Use of Landsat ETM plus data for delineation of water bodies in hilly zones. J Hydroinf 2011;13(4):661-671. |
| **Flow model** | (4) Bierkens MFP, Van Bakel PJT, Wesseling JG. Comparison of two modes of surface water control using a soil water model and surface elevation data. Geoderma 1999;89(1-2):149-175. |
| **Sediment yield** | (5) Boardman J, Foster IDL. The potential significance of the breaching of small farm dams in the Sneeuberg region, South Africa. Journal of Soils and Sediments DEC 2011;11(8):1456-1465. |
| **Not about dam** | (6) Brainwood M, Burgin S, Byrne M. The impact of small and large impoundments on freshwater mussel distribution in the Hawkesbury-Nepean River, southeastern Australia. River research and applications 2008;24(10):1325-1342. |
| **Flow model** | (7) Bryan BA, Higgins A, Holland K, King D, Nolan M, Bjornsson T, Kirby M, et al. Ecohydrological and socioeconomic integration for the operational management of environmental flows. Ecol Appl 2013;23(5):999-1016. |
| **Flow model** | (8) Castro-Orgaz O. Potential Flow Solution for Open-Channel Flows and Weir-Crest Overflow. J Irrig Drain Eng 2013;139(7):551-559. |
| **Dam impact** | (9) Choi S, Lee H, Yoon B, Woo H. Change in Stream Morphology after Gongneung Weir 2 Removal. 한국수자원학회논문집 2009;42(5):425-432. |
| **Dam impact** | (10) Csiki SJC, Rhoads BL. Influence of four run-of-river dams on channel morphology and sediment characteristics in Illinois, USA. Geomorphology FEB 1 2014;206:215-229. |
| **Dam impact** | (11) Csiki S, Rhoads BL. Hydraulic and geomorphological effects of run-of-river dams. Prog Phys Geogr DEC 2010;34(6):755-780. |
| **Historical** | (12) Cubizolle H, Tourman A, Argant J, Porteret J, Oberlin C, Serieyssol K. Origins of European biodiversity: palaeo-geographic signification of peat inception during the Holocene in the granitic eastern Massif Central (France). Landscape Ecol APR 2003;18(3):227-238. |
| **Not about dam** | (13) Cuttle SP, Mason DJ. A flow-proportional water sampler for use in conjunction with a V-notch weir in small catchment studies. Agric Water Manage 1988;13(1):93-99. |
| **Dam impact** | (14) Doyle MW, Stanley EH, Orr CH, Selle AR, Sethi SA, Harbor JM. Stream ecosystem response to small dam removal: Lessons from the Heartland. Geomorphology OCT 1 2005;71(1-2):227-244. |
| **Nutrient dynamics** | (15) Doyle MW, Stanley EH. Exploring potential spatial-temporal links between fluvial geomorphology and nutrient-periphyton dynamics in streams using simulation models. Ann Assoc Am Geogr DEC 2006;96(4):687-698. |
| **Historical** | (16) Elder JT, Gilmour DM, Butler VL, Campbell SK, Steingraber A. On the Role of Coastal Landscape Evolution in Detecting Fish Weirs: A Pacific Northwest Coast Example From Washington State. The Journal of Island and Coastal Archaeology 2014;9(1):45-71. |
| **Dam impact** | (17) Evans JE, Huxley JM, Vincent RK. Upstream channel changes following dam construction and removal using a GIS/remote sensing approach. J Am Water Resour Assoc JUN 2007;43(3):683-697. |
| **Sediment yield** | (18) Feiznia S, Ahmadi H, Jalili SY, Fatahi MA, Abbasi M. Investigation on volume and statistical parameters of sedimentology in upstream channels of Tehran - Qazvin highway located in Khor-Sefidarak Basin. Iranian Journal of Range and Desert Research 2012;19(2):244-263. |
| **Not about dam** | (19) Feld CK, Birk S, Bradley DC, Hering D, Kail J, Marzin A, et al. From Natural to Degraded Rivers and Back Again: A Test of Restoration Ecology Theory and Practice. Advances in Ecological Research, Vol 44 2011;44:119-209. |
| **Dam impact** | (20) Gangloff MM, Hartfield EE, Werneke DC, Feminella JW. Associations between small dams and mollusk assemblages in Alabama streams. J N Am Benthol Soc DEC 2011;30(4):1107-1116. |
| **Dam construction** | (21) Henschel S. The involvement of the private sector to ensure sustainability of systems. Water Practice & Technology 2010;5(4):111-111. |
| **Historical** | (22) Herget J. Holocene development of the River Lippe Valley, Germany: A case study of anthropogenic influence. Earth Surf Process Landforms MAR 2000;25(3):293-305. |
| **Not about dam** | (23) Heritage GL. Drought conditions and sediment transport in the Sabie River. Koedoe 1995;38(2):1-9. |
| **LWD** | (24) Hester ET, Doyle MW, Poole GC. The influence of in-stream structures on summer water temperatures via induced hyporheic exchange. Limnol Oceanogr 2009;54(1):355-367. |
| **Aquifer formation** | (25) Hiller T, Romanov D, Kaufmann G, Epting J, Huggenberger P. Karstification beneath the Birs weir in Basel/Switzerland: A 3D modeling approach. Journal of Hydrology JUL 2 2012;448:181-194. |
| **Historical** | (26) Hoshikawa K, Kobayashi S. Effects of topography on the construction and efficiency of earthen weirs for rice irrigation in Northeast Thailand. Paddy and water environment 2009;7(1):17-25. |
| **Sediment yield** | (27) James LA. Sediment from hydraulic mining detained by Englebright and small dams in the Yuba basin. Geomorphology OCT 1 2005;71(1-2):202-226. |
| **Sediment yield** | (28) Jen C, Lin J, Hsu M, Petley DN. Fluvial transportation and sedimentation of the Fu-shan small experimental catchments. Quaternary international 2006;147(1):34-43. |
| **Evaluation of earthquake** | (29) Kamanbedast MI, Azmoudeh AAE, Hossein M. Dynamic analysis of concrete dam due to seismic forces. World Applied Sciences Journal 2012;17(8):1046-1053. |
| **Dam impact** | (30) Kibler K, Tullos D, Kondolf M. Evolving Expectations of Dam Removal Outcomes: Downstream Geomorphic Effects Following Removal of a Small, Gravel-Filled Dam. J Am Water Resour Assoc APR 2011;47(2):408-423. |
| **Sediment yield** | (31) Mahmoudzadeh A, Erskine WD, Myers C. Sediment yields and soil loss rates from native forest, pasture and cultivated land in the Bathurst area, New South Wales. Australian Forestry 2002;65(2):73-80. |
| **Not about dams** | (32) Marsden JE, Evans JE, Gottgens JF. Dam removals and river channel changes in northern Ohio: implications for Lake Erie sediment budgets and water quality. J Great Lakes Res 2007;33(SI2):87-193. |
| **Sediment yield** | (33) Mousavi SF, Samadi-Boroujeni H. Evaluation of sedimentation in small dam reservoirs in Chaharmahal-Bakhtiary region. Iranian Journal of Science and Technology 1998;22(4):421-429. |
| **Flow model** | (34) Nasri S, Cudennec C, Albergel J, Berndtsson R. Use of a geomorphological transfer function to model design floods in small hillside catchments in semiarid Tunisia. Journal of hydrology 2004;287(1-4):197-213. |
| **Sediment yield** | (35) Ndomba PM. Validation of PSIAC model for sediment yields estimation in ungauged catchments of Tanzania. International Journal of Geosciences 2013;4(7):1101-1115. |
| **Dam impact** | (36) Orr CH, Rogers KL, Stanley EH. Channel morphology and P uptake following removal of a small dam. J N Am Benthol Soc SEP 2006;25(3):556-568. |
| **Dam impact** | (37) Reid HE, Brierley GJ, Mcfarlane K, Coleman SE, Trowsdale S. The role of landscape setting in minimizing hydrogeomorphic impacts of flow regulation. International Journal of Sediment Research MAR 2013;28(2):149-161. |
| **Wetland restoration** | (38) Riddell ES, Lorentz S, Kotze D. The hydrodynamic response of a semi-arid headwater wetland to technical rehabilitation interventions. Water S A 2012;38(1):55-66. |
| **Dam impact** | (39) Roberts SJ, Gottgens JF, Spongberg AL, Evans JE, Levine NS. Assessing potential removal of low-head dams in urban settings: an example from the Ottawa River, NW Ohio. Environ Manage 2007;39(1):113-124. |
| **LWD** | (40) Ryan SE, Bishop EL, Daniels JM. Influence of large wood on channel morphology and sediment storage in headwater mountain streams, Fraser Experimental Forest, Colorado. Geomorphology 2014;217:73-88. |
| **Dam impact** | (41) Salant NL, Schmidt JC, Wilcock PR, Budy P. Unintended consequences of restoration: Loss of riffles and gravel substrates following weir installation. J Environ Manage 2012;109:154-163. |
| **Dam construction** | (42) Salih SA, Kadim LS, Qadir M. Hydrochemistry as indicator to select the suitable locations for water storage in Tharthar valley, Al-Jazira Area, Iraq. Journal of Water Resource and Protection 2012;4(8):648-656. |
| **Flow model** | (43) Sarkar S, Rai RK. Flood Inundation Modeling Using Nakagami-m Distribution Based GIUH for a Partially Gauged Catchment. Water Resour Manage 2011;25(14):3805-3835. |
| **Dam impact** | (44) Sawaske SR, Freyberg DL. A comparison of past small dam removals in highly sediment-impacted systems in the U.S. Geomorphology MAY 15 2012;151:50-58. |
| **Dam impact** | (45) Skalak K, Pizzuto J, Hart DD. Influence of Small Dams on Downstream Channel Characteristics in Pennsylvania and Maryland: Implications for the Long-Term Geomorphic Effects of Dam Removal. J Am Water Resour Assoc FEB 2009;45(1):97-109. |
| **Dam impact** | (46) Škarpich V, Hradecký J, Dušek R. Complex transformation of the geomorphic regime of channels in the forefield of the Moravskoslezské Beskydy Mts.: Case study of the Morávka River (Czech Republic). Catena 2013;111:25-40. |
| **Historical** | (47) Srikantia S. The Talkad sand dunes in Mysore district of Karnataka - their description, evolution and origin. Journal of the Geological Society of India 1997;50(3):315-321. |
| **Dam impact** | (48) Stanley EH, Luebke MA, Doyle MW, Marshall DW. Short-term changes in channel form and macro invertebrate communities following low-head dam removal. J N Am Benthol Soc MAR 2002;21(1):172-187. |
| **Dam impact** | (49) Thoms MC, Walker KF. Channel changes related to low-level weirs on the River Murray, South Australia *in* Carling PA. Lowland floodplain rivers: geomorphological perspectives 1992: 235-249pp. |
| **Flow model** | (50) Thomas RE, Parsons DR, Sandbach SD, Keevil GM, Marra WA, Hardy RJ, et al. An experimental study of discharge partitioning and flow structure at symmetrical bifurcations. Earth Surf Process Landforms 2011;36(15):2069-2082. |
| **Dam impact** | (51) Tullos DD, Finn DS, Walter C. Geomorphic and Ecological Disturbance and Recovery from Two Small Dams and Their Removal. Plos One SEP 18 2014;9(9):e108091. |
| **Sediment yield** | (52) Walker J. The use of sediment modelling techniques to address the differing needs of management on the River Eden, Cumbria, UK. Journal of the Institution of Water and Environmental Management 2001;15(4):252-257. |
| **Not about dam** | (53) Won SM. Distribution of Geomorphological Landscape Resources of Goryeong-gun, and Its Application Plan. 한국지역지리학회지 2008;14(4):279-289. |
| **Not about dam** | (54) Woo H, Han MS, Kim CW. Situation and Prospect of Ecological Engineering for Stream Restoration in Korea. KSCE Journal of Civil Engineering 2005;9(1):19-27. |
